# The role of ^18^F-FDG PET/CT in patients with synchronous multiple primary malignant neoplasms occurring at the same time

**DOI:** 10.3389/fonc.2022.1068055

**Published:** 2022-12-02

**Authors:** Zhe Huang Luo, Wan Ling Qi, Ai Fang Jin, Feng Xiang Liao, Qian Liu, Qing Yun Zeng

**Affiliations:** ^1^ Department of Nuclear Medicine, Jiangxi Provincial People’s Hospital (The First Affiliated Hospital of Nanchang Medical College), Nanchang, China; ^2^ Department of Pathology, Jiangxi Provincial People’s Hospital (The First Affiliated Hospital of Nanchang Medical College), Nanchang, China

**Keywords:** synchronous multiple primary malignant neoplasms, positron emission tomography/computed tomography, ^18^F-fluorodeoxyglucose (^18^F-FDG), conventional imaging, diagnostic performance

## Abstract

**Background:**

Synchronous multiple primary malignant neoplasms occurring at the same time (SMPMNS) are not currently uncommon in clinical oncological practice; however, the diagnostic performance of ^18^F-fluorodeoxyglucose positron emission tomography/computed tomography (^18^F-FDG PET/CT) for SMPMNS needs further elucidation.

**Purpose:**

This study aimed to evaluate the application of ^18^F-FDG PET/CT in patients with SMPMNS.

**Materials and methods:**

The clinical and imaging data of 37 patients with SMPMNS who had undergone ^18^F-FDG PET/CT from October 2010 to December 2020 were retrospectively analyzed. The kappa consistency test was applied to evaluate the consistency of the diagnostic performance between PET/CT and conventional imaging (CI). The sensitivity, specificity, and accuracy of PET/CT and CI in the detection of metastatic lesions were compared.

**Results:**

This retrospective diagnostic study included 74 lesions identified in 37 patients with SMPMNS, with 94.6% of patients having double primary tumors. Of the incidences of SMPMNS, 18.9% occurred in the same organ system, with respiratory tumors being the most common type of neoplasm (43.2%) and the lung being the most common primary site (40.5%). The overall survival of SMPMNS patients without metastases was longer than that of those with metastases (*χ* ^2^ = 12.627, *p* = 0.000). The maximum standardized uptake value (SUV_max_), the SUV_max_ ratio (larger SUV_max_/smaller SUV_max_), and the difference index of SUV_max_ (DISUV_max_) [(larger SUV_max_ − smaller SUV_max_)/larger SUV_max_] of the primary lesions ranged from 0.9 to 41.7 (average = 12.3 ± 7.9), from 0.3 to 26.7 (average = 4.4 ± 6.9), and from 0.0% to 96.3% (average = 50.3% ± 29.3%), respectively. With regard to diagnostic accuracy, PET/CT and CI showed poor consistency (*κ* = 0.096, *p* = 0.173). For the diagnosis of primary lesions (diagnosed and misdiagnosed), PET/CT and CI also showed poor consistency (*κ* = 0.277, *p* = 0.000), but the diagnostic performance of PET/CT was better than that of CI. In the diagnosis of metastases, the patient-based sensitivity, specificity, and accuracy of PET/CT were 100.0%, 81.8%, and 89.2%, respectively, while those of CI were 73.3%, 100.0%, 89.2%, respectively. The sensitivity and specificity values were significantly different, with PET/CT having higher sensitivity (*p* = 0.02) and CI showing higher specificity (*p* = 0.02).

**Conclusions:**

^18^F-FDG PET/CT improves the diagnostic performance for SMPMNS and is a good imaging modality for patients with SMPMNS.

## Introduction

Multiple primary malignant neoplasms (MPMNs) are defined as two or more unrelated primary malignant neoplasms that occur simultaneously or successively in one or more organs of the same host (1). They are generally diagnosed according to the criteria established by Warren and Gates (2) and are classified as synchronous MPMN (SMPMN) or metachronous MPMN (MMPMN) depending on the interval between the diagnosis of the first and second primary tumors (3), i.e., SMPMN when the second tumor was identified at the same time (SMPMNS) or successively within 6 months after the diagnosis of the first tumor and MMPMN when the second tumor was identified at an interval of more than 6 months. MPMNs can originate from any site, such as the same organ or paired organs (POs). According to published literature on different countries or districts, the reported incidences of MPMNs vary between 0.5% and 11.7% (0.5%–3.7% in China and 0.7%–11.7% in other countries) (4–6) and have been increasing during the last decade in China. The early diagnosis and appropriate assessment of SMPMNS can alter the therapeutic strategy and improve the overall prognosis. Conventional imaging (CI) techniques, including ultrasound (US), computed tomography (CT), magnetic resonance imaging (MRI), and nuclear imaging, have clear limitations due to their regional imaging modality in the detection of SMPMNS.

Positron emission tomography/computed tomography (PET/CT) has been widely used for the diagnosis, staging, restaging, recurrence, and the effective evaluation of tumors. Because of the integrated imaging modality of anatomic and functional imaging and whole-body scanning, PET/CT may have some advantages over CI in the detection of SMPMNS. At present, there are only a few PET/CT studies on SMPMNS. This study aimed to evaluate the role of ^18^F-fluorodeoxyglucose PET/CT (^18^F-FDG PET/CT) in the diagnosis of SMPMNS.

## Materials and methods

This study was approved by our institution’s Ethics Review Board. Patient written informed consent was waived owing to the retrospective design of the study. MPMNs were diagnosed according to the criteria established by Warren and Gates (2).

### Patients

The patients included in this study met the following criteria: 1) had undergone ^18^F-FDG PET/CT in our hospital from October 2010 to December 2020; 2) had two or more malignant neoplasms at the same time (SMPMNS) shown in the PET/CT scan; 3) had complete medical data for basic patient characteristics such as age, gender, and histological type of the primary tumor; 4) SMPMNS were confirmed by biopsy/surgical histopathology (and immunohistochemistry) within 2 weeks after PET/CT; and 5) had an ordinary occupation and denied having a history of exposure to radioactive or toxic substances. The exclusion criteria were as follows: 1) both or more primary tumors of SMPMNS were histopathologically confirmed before PET/CT and 2) patients who had received radiochemotherapy for primary tumor before PET/CT.

**Figure 1:**
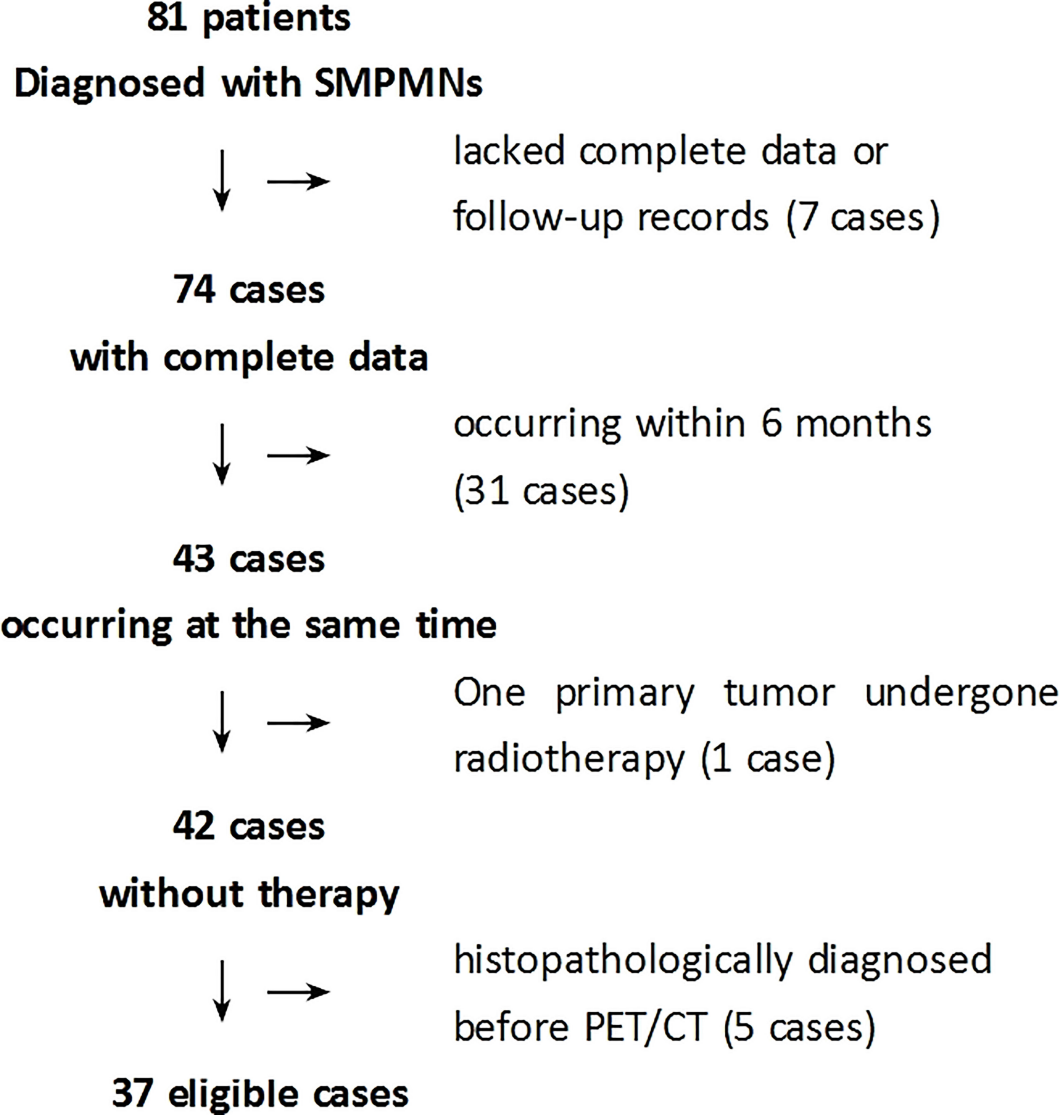
Flowchart of the selection of eligible patients. *SMPMNs*, synchronous multiple primary malignant neoplasms.

The number of cases undergoing ^18^F-FDG PET/CT in our center during the study period determined the sample size (**Figure 1**).

### Data acquisition and reconstruction of ^18^F-FDG PET/CT

^18^F-FDG PET/CT imaging was performed using a GE Discovery STE PET/CT scanner. Patients fasted for at least 4 h before ^18^F-FDG injections, and scans were obtained 50–60 min after intravenous administration of ^18^F-FDG (5.5 MBq/kg). Non-contrast CT data were used for the anatomical correlation and attenuation correction of the PET images. The CT data were acquired with the following settings: 120 kV; 100–140 mA; pitch, 1.75:1; collimation, 16 × 3.75 mm; and rotation cycle, 0.5 s. Whole-body PET scans were acquired in 3D mode and performed from the vault of the skull to the mid-thigh, with 3 min per bed position acquisition time.

### Image interpretation and comparison of the diagnostic performance of PET/CT and CI

**Figure 2:**
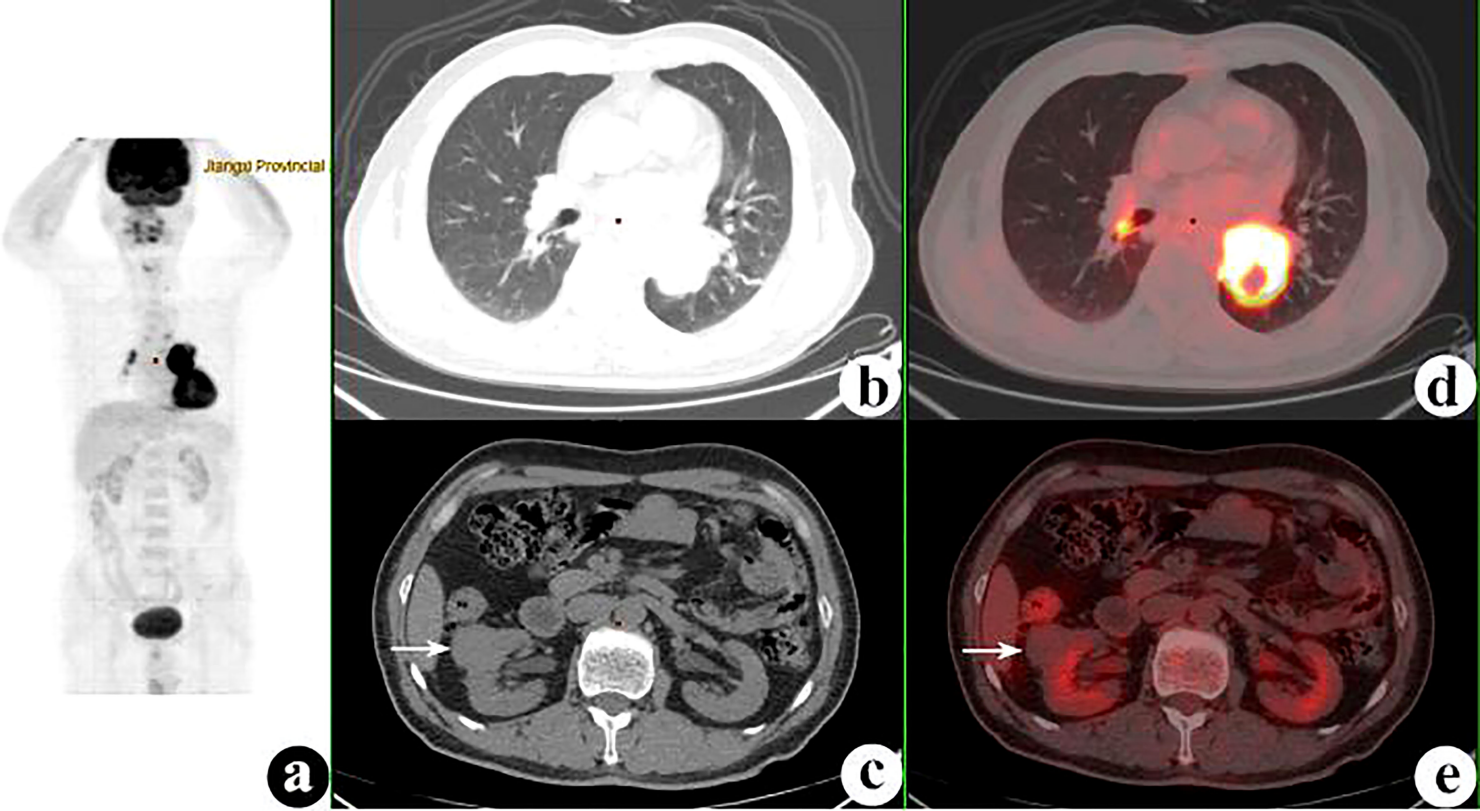
^18^F-fluorodeoxyglucose positron emission tomography/computed tomography (^18^F-FDG PET/CT) of a 54-year-old man with lung squamous cell carcinoma and renal small cell carcinoma. PET/CT demonstrated a 52 × 47-mm mass with a maximum standardized uptake value (SUV_max_) of 15.5 in the left lower lung and a 30 × 30-mm nodule with SUV_max_ of 1.1 (*white arrows*) in the right kidney. Several hilar and mediastinal lymph nodes with different FDG uptake levels were proven to be hyperplasia. **(A)** PET maximum intensity projection (MIP). **(B, C)** Axial CT. **(D, E)** Fusion images.

**Figure 3:**
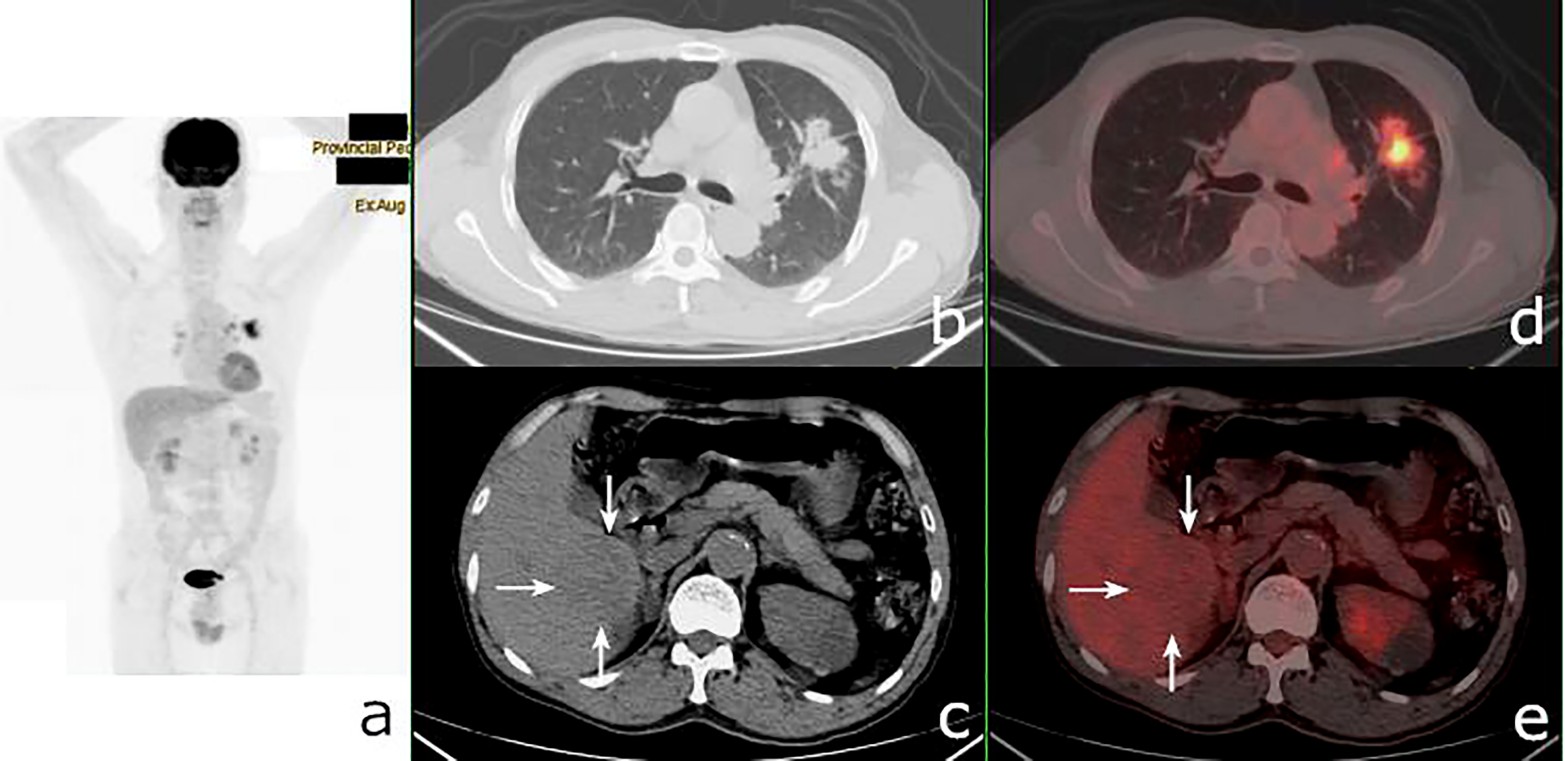
^18^F-fluorodeoxyglucose positron emission tomography/computed tomography (^18^F-FDG PET/CT) of a 62-year-old man with lung adenocarcinoma and hepatocellular carcinoma. PET/CT revealed a lobulated mass with central FDG uptake (maximum standardized uptake value, SUV_max_ = 8.9) and a 48 × 49-mm hypodense mass with SUV_max_ = 2.0 in the right hepatic lobe (*white arrows*). **(A)**: PET maximum maximum intensity projection (MIP). **(B, C)**: Axial CT. **(D, E)** Fusion images.

**Figure 4:**
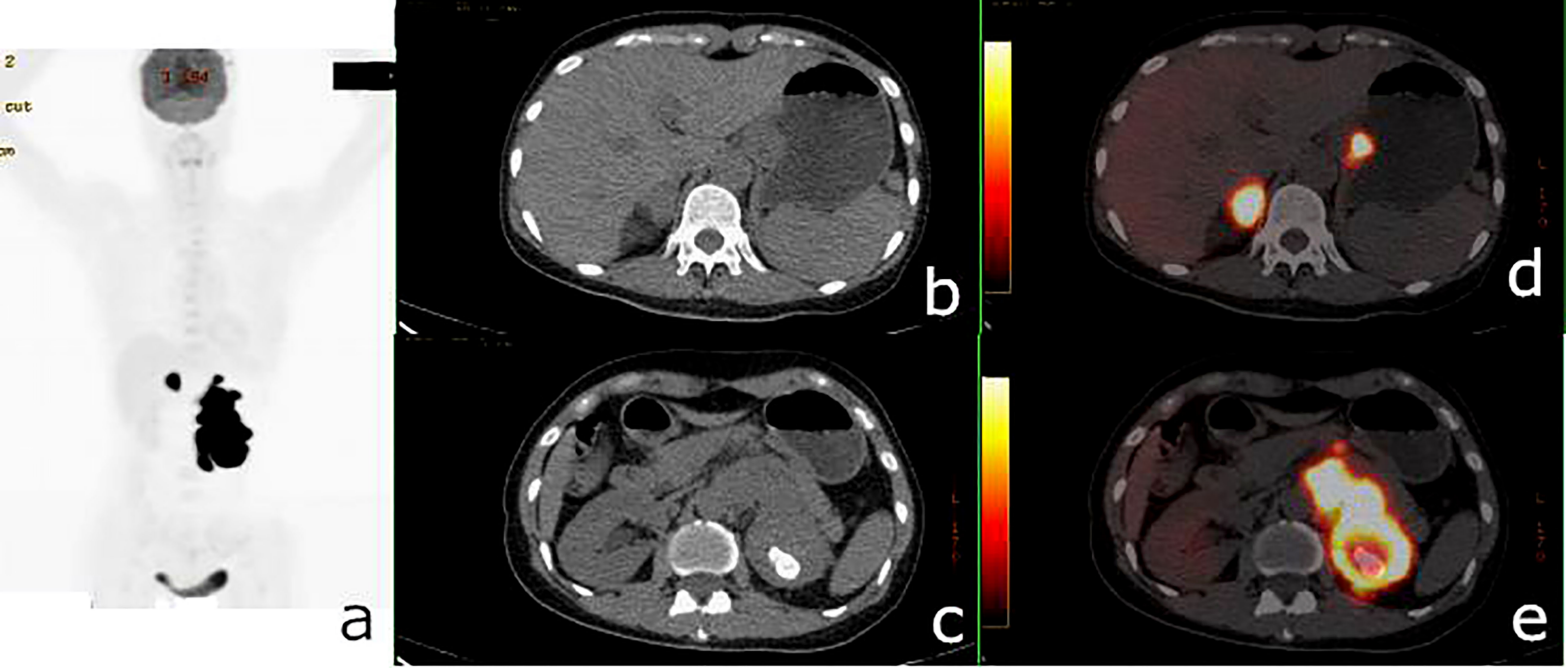
^18^F-fluorodeoxyglucose positron emission tomography/computed tomography (^18^F-FDG PET/CT) of a 50-year-old woman with renal adenocarcinoma and gastric stromal tumor. PET/CT demonstrated an irregular mass in the left kidney with a maximum standardized uptake value (SUV_max_) of 45.3 and a 24 × 19-mm nodule in the gastric cardia with SUV_max_ = 28.3. A hypermetabolic right adrenal nodule and several hypermetabolic retroperitoneal lymph nodes were also shown. **(A)** PET maximum intensity projection (MIP). **(B, C)** Axial CT. **(D, E)** Fusion images.

**Figure 5:**
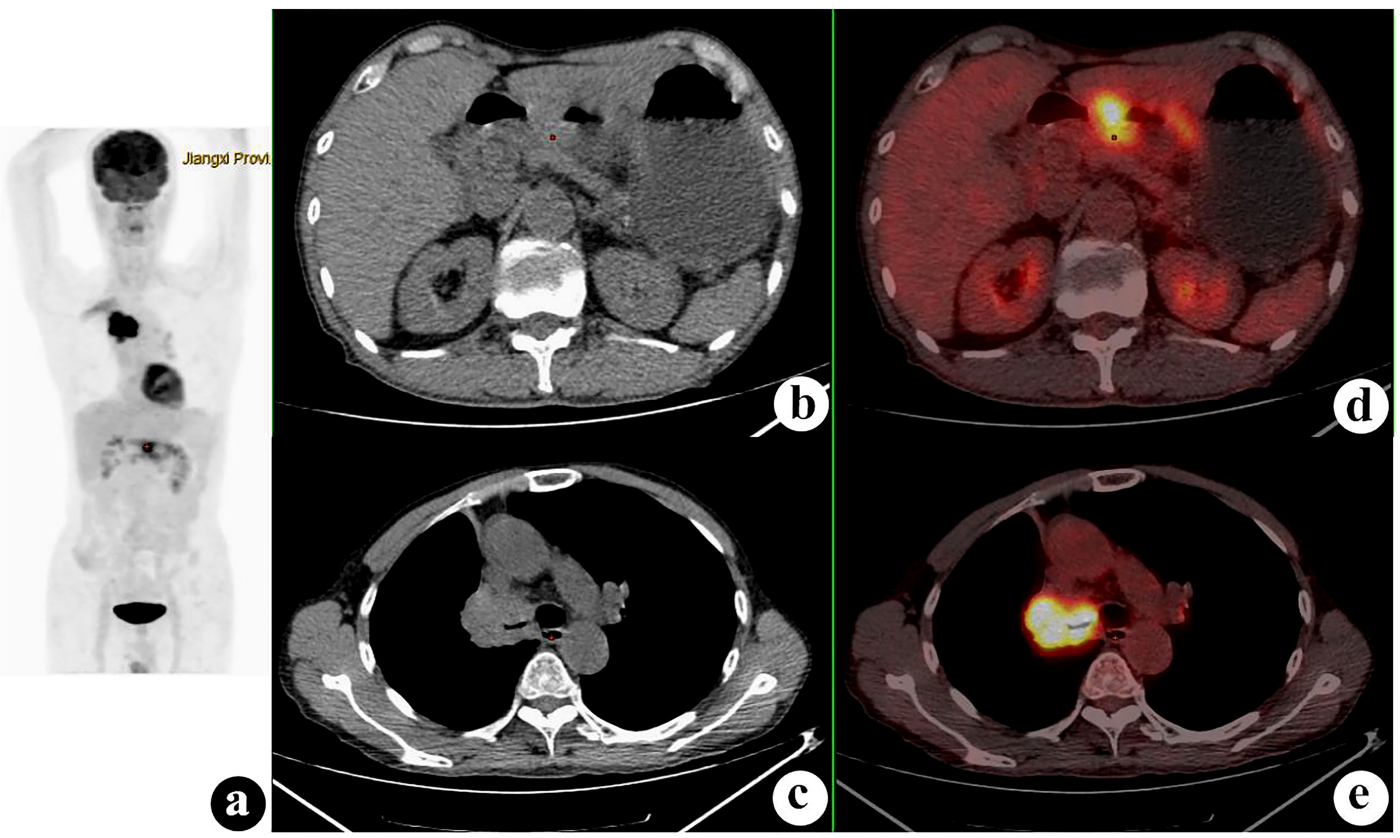
^18^F-fluorodeoxyglucose positron emission tomography/computed tomography (^18^F-FDG PET/CT) of a 60-year-old man with gastric adenocarcinoma (GA) and synchronous right lung squamous cell carcinoma. GA was proven before PET/CT, and PET/CT demonstrated a 47 × 40-mm right hilar mass with a maximum standardized uptake value (SUV_max_) of 13.9. **(A)** PET maximum intensity projection (MIP). **(B, C)** Axial CT. **(D, E)** Fusion images.

**Figure 6:**
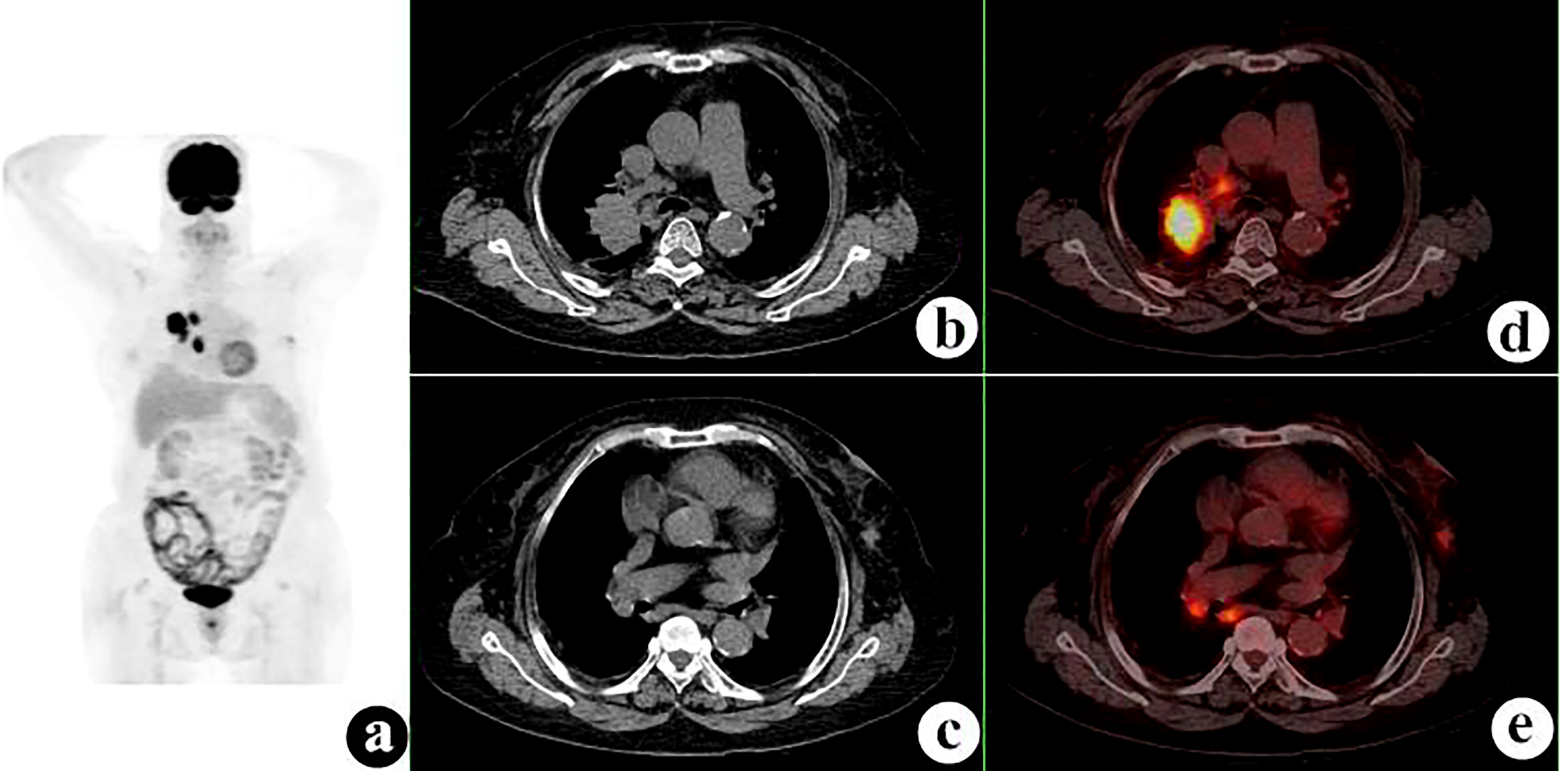
^18^F-fluorodeoxyglucose positron emission tomography/computed tomography (^18^F-FDG PET/CT) of a 78-year-old woman with lung squamous cell carcinoma, synchronous left breast ductal carcinoma, and lymph node metastases. PET/CT demonstrated a 32 × 45-mm mass with a maximum standardized uptake value (SUV_max_) of 12.2 in the right upper lobe, a 15 × 9-mm nodule with SUV_max_ = 2.6 in the left breast, and several mediastinal lymph nodes with SUV_max_ = 10.2. **(A)** PET maximum intensity projection (MIP). **(B, C)** Axial CT. **(D, E)** Fusion images.

The clinical information and PET/CT imaging data of all patients with SMPMNS were retrospectively analyzed. All focal uptakes greater than the background that could not be explained by the physiological uptake were considered as indicative of lesions. The diagnosis of SMPMNS with ^18^F-FDG PET/CT was based on the following: 1) the maximum standardized uptake values (SUV_max_) of the two suspected tumor lesions were significantly different (**Figures 2**, **3**); 2) two suspected tumor lesions occurred at different sites or organs with very few tumor metastasis to each other (**Figures 4**, **5**); and 3) one tumor was confirmed and the other suspected tumor lesion did not match the characteristics of common metastases (**Figure 6**).

The presence of SMPMNS and metastatic lesions in the ^18^F-FDG PET/CT results was carefully recorded, including the site, shape, edge, size, density, and the SUV_max_ of each lesion. Misdiagnosed and missed primary tumors on ^18^F-FDG PET/CT were retrieved and reviewed by two board-certified radiologists who served for 30 (ZL) and 8 (WQ) years in radiology departments. The SUV_max_ ratio, the difference in the SUV_max_ (ΔSUV_max_), and the difference index of SUV_max_ (DISUV_max_) of the concomitant tumors were calculated as follows:


$$SUV_{\max}\;ratio=\frac{L\arg er\;SUV_{\max}}{Smaller\;SUV_{\max}}$$

$$\Delta SUV_{\max}=L\arg er\;SUV_{\max}-Smaller\;SUV_{\max}$$

$$DISUV_{\max}=\frac{\Delta SUV_{\max}}{L\arg er\;SUV_{\max}}\times 100\%$$

The SMPMNS and metastases reported using CI (including CT, MRI, US, and scintigraphy), performed within 15 days prior to ^18^F-FDG PET/CT, were also carefully documented.

CI is a common detection method for SMPMNS. The consistency in the diagnostic performance of PET/CT and CI was examined, and the sensitivity, specificity, and accuracy of both methods in the detection of metastatic lesions were compared.

### Follow-ups

All patients who completed PET/CT examinations at our institution were routinely followed up. The patients in this study have been followed up for 6–36 months (average = 24.8 ± 9.7 months) after PET/CT examination. The follow-up methods included outpatient examination, telephone or web chat follow-up, and assessment of inpatient medical records. Overall survival was calculated from the date of diagnosis of SMPMNS to the date of death of patients or the date of last follow-up.

### Statistical analysis

The SPSS 19.0 software package for PC was used for statistical processing of the obtained data. Categorical variables were expressed as frequencies or percentages, while numerical variables were expressed as the mean ± standard deviation (SD). Statistical comparison of the categorical variables was performed using McNemar’s test. A log-rank test was performed to evaluate the differences in the survival rates of SMPMNS patients with and without metastasis. *P*-values less than 0.05 were considered significant. A consistency test was also performed to evaluate the diagnostic results between the ^18^F-FDG PET/CT and CI methods, with the evaluation criteria for the kappa values as follows: *κ* ≥ 0.75 indicates good consistency in the diagnostic results; 0.4 ≤ *κ*< 0.75 indicates general consistency in the diagnostic results; and *κ*< 0.4 indicates poor consistency in the diagnostic results.

## Results

### Clinical features of eligible patients

Between October 2010 and December 2020, a total of 81 patients with SMPMNs underwent ^18^F-FDG PET/CT. Of these, 44 patients were excluded base on the inclusion and exclusion criteria. The remaining 37 patients with complete follow-up data and who met the enrollment criteria (**Figure 1**) were finally included in the study, with 24 men (24/37, 64.9%) and 13 women (13/37, 35.1%) aged 19–82 years (average = 65.4 ± 11.6 years). The demographic and clinical information of the patients are summarized in **Table 1**.

**Table 1:** Clinical features and ^18^F-fluorodeoxyglucose positron emission tomography/computed tomography (^18^F-FDG PET/CT) data of patients with synchronous multiple primary malignant neoplasms occurring at the same time (SMPMNS).

Case no.	Sex	Age (years)	PMN1	SUV_max_	PMN2	SUV_max_	Metastasis/(adjacent invasion)	Misdiagnosis or missed diagnosis
1	F	49	Endometrial carcinoma	2.7	Ureteral squamous cell carcinoma	7.7		Failed 1, MD2
2	M	67	Epiglottis squamous cell carcinomas	15.9	Lung adenocarcinoma	1.8	LN	
3	M	60	Prostate cancer	13.5	Lung squamouscell carcinomas	5.7	Brain, LN, (SV)	
4	M	74	Colon adenocarcinoma	12.3	Lung squamous cell carcinomas	8.4	LN, liver	
5	M	62	Lung adenocarcinoma	8.9	Hepatocellular carcinoma	2.0		
6	M	54	Malignant pleural mesothelioma	9.4	Lung adenocarcinoma	0.8		
7	M	65	Lung squamous cell carcinomas	10.4	Prostate cancer	10.7	Bone, LN	
8	M	78	Lung squamous cell carcinomas	10.6	Gastric stromal tumor	6.1		
9	F	78	Lung squamous cell carcinomas	12.2	Breast ductal carcinoma	2.6	LN	
10	F	72	Bladder cancer	23.9	Anal canal adenocarcinoma	6.7	LN	Missed 2
11	M	69	Lung squamous cell carcinomas	4.5	Lung adenocarcinoma	0.9		Missed 2
12	M	54	Renal small cell carcinoma	1.1	Lung squamous cell carcinomas	15.5	LN^d^	
13	M	72	Maxillofacial basal cell carcinoma	4.3	Lung adenocarcinoma	4.2		
14	F	74	Breast ductal carcinoma	3.2	Lung adenocarcinoma	6.8		
15^a^	F	50	Renal adenocarcinoma	22.5	Gastric stromal tumor	17.7	Ad, LN	
16	M	60	Gastric adenocarcinoma	11.8	Lung squamous cell carcinomas	13.9		
17	M	55	Lung squamous cell carcinomas	29.8	Lung adenocarcinoma	1.1	Bone, LN	MD 2
18	M	58	Cerebral glioblastoma	23.8	Colon adenocarcinoma	15.9		
19^b^	F	65	Lung squamous cell carcinomas	7.2	Colon adenocarcinoma	9.9		
20	M	64	Lung adenocarcinoma	13.2	Renal clear cell carcinoma	1.0		
21	F	74	Vulval skin melanoma	5.3	Renal Clear cell carcinoma	1.1		Fail 1
22	M	64	Lung squamous cell carcinomas	18.2	Small salivary gland carcinoma	22.2		
23	F	70	Lung adenocarcinoma	16.1	Renal clear cell carcinoma	4.7	Lung, liver, bone, LN	
24	M	61	Lung squamous cell carcinomas	4.6	Prostate cancer	16.4	(SV)	
25	M	59	Oral squamous cell carcinomas	8.3	Esophageal squamous cell carcinomas	6.8		
26	F	82	Papillary thyroid carcinoma	4.8	Lung squamous cell carcinomas	6.6	LN^d^	
27	M	63	Gastric adenocarcinoma	12.2	Small Intestine adenocarcinoma	15.6	Ad, lung	MD 2
28	M	73	Gastric adenocarcinoma	3.7	Lung adenocarcinoma	9.7	Ad, LN, brain	
29	M	75	Lung squamous cell carcinomas	9.1	Prostate cancer	7.1	Ad^d^ (rectum, SV)	
30	F	79	Lung adenocarcinoma	7.6	Papillary thyroid carcinoma	10.3	bone^d^	
31	M	70	Lung squamous cell carcinomas	19.5	Small Intestine adenocarcinoma	14.8	LN, lung	MD 2
32	F	64	rectal adenocarcinoma	41.7	Ductal carcinoma of breast	7.7	Bone, lung	
33	F	70	Gastric adenocarcinoma	2.8	Lung adenocarcinoma	5.4		
34	M	82	Lung squamous cell carcinomas	23.2	Cutaneous diffuse large B-cell lymphoma	6.6		MD 2
35	M	64	Esophageal squamous cell carcinomas	16.5	Lung squamous carcinomas	17.4	Bone, LN	
36	M	70	Lung squamous cell carcinomas	11.5	Thyroid clear cell carcinoma	4.2	Lung	
37	F	19	Gastric adenocarcinoma^c^	19.4	Gastric diffuse large B-cell lymphoma^c^	19.4		Missed 2

SMPMNS, synchronous multiple primary malignant neoplasms occurring at the same time; PMN, primary malignant neoplasm; SUV_max_, maximum standardized uptake value; M, male; F, female; LN, lymph node; Ad, adrenal gland; SV, seminal vesicle gland; Failed 1, failed to report tumor 1 that had been diagnosed before PET/CT; MD 1 or 2, misdiagnosed primary tumor 1 or 2 as metastasis; Miss 2, missed tumor 2

a More than 11 years after the resection of gastric cancer.

b Less than 6 months after surgery of scalp squamous cell carcinomas.

c Could not be distinguished on PET/CT and the SUV_max_ of the two diseases considered as the same.

d Excludes metastasis.

From all 37 patients, a total of 74 malignant tumors were identified when they were diagnosed with SMPMNS within 2 weeks after PET/CT. Two patients (5.4%) had triple primary malignancies: one underwent surgery for gastric cancer 11 years ago, fulfilling the MMPMN and SMPMN criteria, and the other underwent surgical resection for scalp squamous cell carcinoma 5 months ago, consistent with the diagnostic criteria of synchronous triple primary malignancy. Both of them denied having a history of chemoradiotherapy after their surgery. The remaining 35 (94.6%) patients all had synchronous double primary malignant tumors. SMPMNS originated in the same organ system in 7 (18.9%) cases: respiratory tumors in 4 (10.8%) and digestive tumors in 3 (8.1%) cases. Of the 74 tumors, respiratory tumors comprised the most common type of neoplasm (32/74, 43.2%), followed by digestive tumors (20/74, 27.0%). The lung was the most common primary site (30/74, 40.5%), followed by the stomach (8/74, 10.8%).

Documented proven metastases were present in 15 (40.5%) cases and adjacent invasion in 3 (8.1%) cases (**Table 2**).

**Table 2:** Metastases and adjacent invasion of patients with synchronous multiple primary malignant neoplasms occurring at the same time (SMPMNS).

		Metastasis (cases)						Adjacent invasion (cases)	
		LN	Bone	Lung	Ad	Brain	Liver	Rectum	SV
^18^F-FDG PET/CT		14	6	4	4	2	2	1	3
Conventional imaging		5	3	3	3	2	3	1	3
Documented (proven by)	Follow-up	9	5	4	3	2	3	1	3
	Biopsy	3	–	–	–	–	–	–	–

LN, lymph node; Ad, adrenal; SV, seminal vesicle gland.

**Figure 7:**
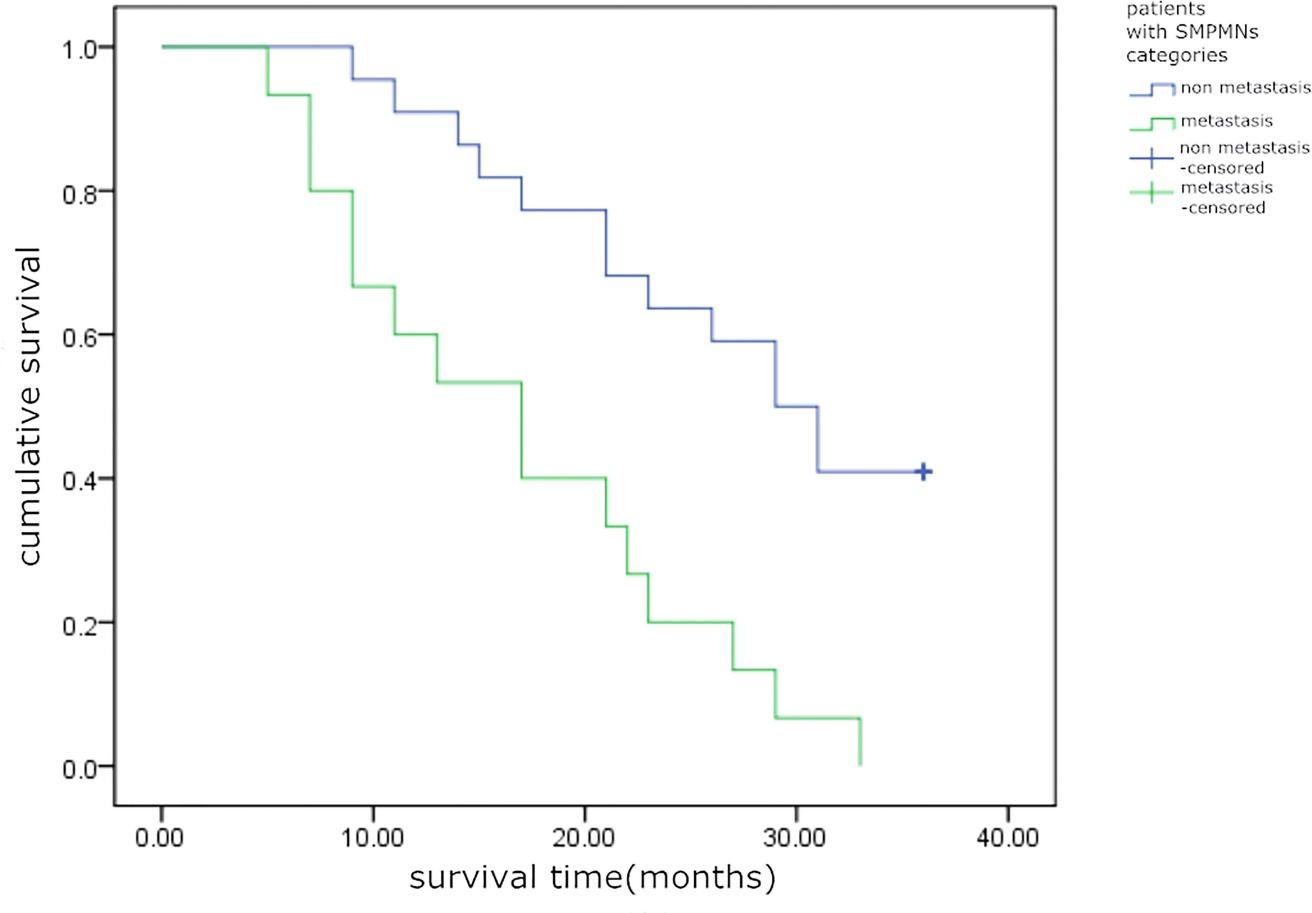
Kaplan–Meier survival analysis, time to death (in months), for the two categories of patients with synchronous multiple primary malignant neoplasms (SMPMNs) (log-rank test, *χ* ^2^ = 12.627, *p* = 0.000).

Differences in the overall survival rates among the SMPMNS patients with and without metastases are demonstrated in **Figure 7**. The log-rank test revealed significant lower survival rates in patients with metastases (*χ* ^2^ = 12.627, *p* = 0.000), with a median time to death of 17 months (95%CI = 9.6–24.4); for SMPMNS patients without metastases, the median time to death was 29 months (95%CI = 23.3–34.7).

### Diagnosed, misdiagnosed, or missed SMPMNS on ^18^F-FDG PET/CT and the SUV_max_ of SMPMNS

Of the 37 patients with SMPMNS, 28 (75.7%) were diagnosed with SMPMNS on the PET/CT report (**Table 3**), one primary tumor was misdiagnosed as metastasis in 5 (13.5%) cases, and diagnosis was missed in 5 (13.5%) cases (including one endometrial carcinoma and one vulval melanoma, both having been histopathologically diagnosed before PET/CT).

**Table 3:** Diagnostic performance for synchronous multiple primary malignant neoplasms occurring at the same time (SMPMNS): ^18^F-fluorodeoxyglucose positron emission tomography/computed tomography (^18^F-FDG PET/CT) vs. conventional imaging.

Case	Conventional imaging prior to PET/CT			^18^F-FDG PET/CT		
	Diagnosed	M and/or AI	MD or missed	Diagnosed	M and/or AI	MD or missed
1			Missed, MD			Missed, MD
2		M	MD	**+**	M	
3		M, AI	MD	**+**	M, AI	
4		M	MD	**+**	M	
5	**+**			**+**		
6			MD	**+**		
7		M	MD	**+**	M	
8			Missed	**+**		
9		M	Missed	**+**	M	
10			Missed		M	Missed
11			Missed			Missed
12	**+**			**+**	M*	
13	**+**			**+**		
14			Missed	**+**		
15		M	Missed	**+**	M	
16			Missed	**+**		
17		M	MD		M	MD
18			MD	**+**		
19	**+**			**+**		
20	**+**			**+**		
21			Missed			Missed
22			Missed	**+**		
23		M	MD	**+**	M	
24		AI	Missed	**+**	AI	
25			Missed	**+**		
26			Missed	**+**	M*	
27		M	MD		M	MD
28		M	Missed	**+**	M	
29		AI	Missed	**+**	M*, AI	
30			Missed	**+**	M*	
31			Missed		M	MD
32			Missed	**+**	M	
33			Missed	**+**		
34			Missed			MD
35		M	Missed	**+**	M	
36			Missed	**+**	M	
37			Missed			Missed

The plus sign means diagnosed with SMPMNS. Patient-based consistency test of the diagnostic performance between PET/CT and conventional imaging for SMPMNS: κ = 0.096, p = 0.173; McNemar test, p = 0.000. Patient-based consistency test for revealing primary tumors (diagnosed + misdiagnosed) between PET/CT and conventional imaging: κ = 0.277, p = 0.000; McNemar test, p = 0.001.

M, metastasis; AI, adjacent invasion; MD, misdiagnosed one primary tumor as a metastasis; missed, missed one primary tumor; M*, exclude metastasis by follow-up.

The 74 tumors had an average SUV_max_ of 12.3 ± 7.9 (range = 0.9–41.7). The primary tumors missed by PET/CT were retrieved and the SUV_max_ recalculated according to the confirmed site. The average SUV_max_ ratio, ΔSUV_max_, and the DISUV_max_ of SMPMNs were 4.4 ± 6.9 (range = 0.3–26.7), 7.2 ± 7.6 (range = 0.0–34.0), and 50.3% ± 29.3% (range = 0.0%–96.3%), respectively. The ΔSUV_max_ values were ≥10.0 in 13 (35.1%) cases (**Figure 2**), 5.0 ≤ ΔSUV_max_< 10.0 in 8 (21.6%) cases (**Figure 3**), and<5 in 16 (43.2%) cases (**Figure 4**).

Two hypermetabolic lesions were found in different organs in which the tumors rarely spread from one another in five cases (cases 1, 10, 15, 21, and 32), with 4 (80%) cases being diagnosed (**Figures 5**, **6**).

Hypermetabolic lesions with suspected metastases were identified in 19 (51.4%) cases, while adjacent invasion was identified in 3 (8.1%) cases (**Tables 1**, **2**).

### Diagnostic performance: CI *VS*. ^18^F-FDG PET/CT

A total of 41 CT scans, 49 ultrasound examinations, 16 MRI, and 6 bone scintigraphy were performed in the 37 patients with SMPMNS before PET/CT imaging. On CI, SMPMNS were reported in 5 (13.5%) cases, while one primary tumor was misdiagnosed as metastasis in 10 (27.0%) cases and missed in 23 (62.2%) cases (**Table 3**). For the correct diagnosis of SMPMNS, ^18^F-FDG PET/CT and CI showed poor consistency (*κ* = 0.096, *p* = 0.173), but a better diagnostic performance in patients with SMPMNS was found using ^18^F-FDG PET/CT. The diagnostic results for revealing primary lesions (diagnosed and misdiagnosed) between ^18^F-FDG PET/CT and CI also showed poor consistency (*κ* = 0.277, *p* = 0.000), but ^18^F-FDG PET/CT was superior to CI.

Metastases and adjacent invasion reported on CI are shown in **Tables 2**, **3**. For the diagnosis of metastasis (not including primary tumors misdiagnosed as metastases), the patient-based sensitivity, specificity, and accuracy values of ^18^F-FDG PET/CT were 100.0%, 81.8%, and 89.2%, respectively, while those of CI were 73.3%, 100.0%, and 89.2%, respectively. PET/CT and CI showed similar accuracy. The differences in the sensitivity and specificity values were significant, with PET/CT showing higher sensitivity (*p* = 0.020) and CI having higher specificity (*p* = 0.020).

After PET/CT examination, the regimens of 14 (37.8%) patients were changed.

## Discussion

In this study, we first evaluated the application of ^18^F-FDG PET/CT in patients with synchronous multiple primary malignant neoplasms occurring at the same time (SMPMNS). It was demonstrated that SMPMMS show some clinical features and that ^18^F-FDG PET/CT has good diagnostic performance for SMPMNS, including making the correct diagnosis and displaying primary tumor lesions and metastatic lesions.

MPMNs are not currently uncommon in clinical oncological practice (7) and have been increasing in incidence (8, 9). Most MPMNs are double primary malignancy, while triple or more primary cases are rare (10, 11). They are usually found in the same organ, POs, or the organ of the identical organ system (OIS) (6, 12, 13). In all MPMNs, the prevalence of SMPMNs was lower than that of MMPMNs (10, 11, 14–16). A study (14) demonstrated that the most common SMPMN sites are the digestive tract organs. SMPMNs can occur at the same time or successively at a 6-month interval. In this series, 35 cases were double primary malignant neoplasms. This result is roughly similar to that of the previous reports (10, 11). However, SMPMNs originated in the same organ, with POs being lesser. Tanjak et al. (17) found that the top 10 SMPMNs are located in breast, colorectal, and head and neck cancer, among others, while the top 10 multiple primary cancer types are also in the top 10 single primary cancers. Our results suggest that the lung is the most common SMPMNS site rather than the breast or digestive canal. This discrepancy may be related to differences in the samples. Lung cancer is the most common malignant tumor in China, and most of those who undergo PET/CT in our center are patients with lung cancer.

More and more MPMNs have been identified clinically due to better cancer screening and detection technology, as well as the improved therapeutic planning for malignancies that leads to improvements in the survival time of patients. The mechanism of the development of MPMNs has been elucidated by a number of relevant studies. It is believed that their occurrence is closely related to genetic predisposition, immunological status, overexposure to carcinogenic factors, and increased life expectancy (12, 13, 18). The average age of patients with SMPMN is usually over 60 years (15). The average age of the patients included in this study was 65 years. All patients had in ordinary occupation and denied a history of exposure to radioactive or toxic substances, and they also denied a history of radiotherapy and chemotherapy, but cancer radial surgery, suggesting that SMPMNS are more likely to arise in the elderly. The study of Tanjak et al. (17) also revealed that patients with MPMN were significantly older than those with a single tumor. In addition, the same study also revealed that there were more women than men with SMPMNs (62.3% *vs*. 37.7%), but our series showed the opposite; SMPMNS affected more men than women (64.9% *vs*. 35.1%). This difference may be a result of the different samples; for example, the SMPMNS in this series all occurred at the same time, not including those SMPMNs that occurred successively in a 6-month interval. Unfortunately, pathogenic gene mutation tests in peripheral blood samples were not performed for most cases in the series. Whether these patients had a genetic mutation remains unclear.

The prognosis of patients with SMPMNS is significantly better than that of patients with single primary tumors and metastases; therefore, it is important to distinguish SMPMNS from single primary tumors with metastases. The diagnosis of SMPMNS is mainly based on histopathology, the immunohistochemistry technique used, and genomic assessments (13, 19). ^18^F-FDG PET/CT imaging has become increasingly important in the diagnosis and clinical management of SMPMNS. Ishimori et al. (20) found that ^18^F-FDG PET/CT detected other unexpectedly primary malignancies with a high fluorodeoxyglucose (FDG) uptake in at least 1.2% of cancer patients. Paolini et al. (21) described a case of diffuse large B-cell lymphoma confirmed by PET/CT-guided bone marrow biopsy in a patient with hair cell leukemia. Similarly, Delin et al. (22) reported a case of synchronous lung bronchoalveolar cell carcinoma and squamous cell carcinoma with significantly different levels of FDG uptake. SUV_max_ plays an important role in differentiating SMPMNS from metastasis. Liu et al. (23) and Dijkman et al. (24) showed that the SUV_max_ ratio (optimal cutoff = 1.7) and DI_SUVmax_ (optimal cutoff = 41%) were beneficial to differentiating synchronous multiple primary lung cancers from intrapulmonary metastasis. In this series, the SUV_max_ ratio and the DI_SUVmax_ of concomitant tumors were 4.4 ± 6.9 and 50.3% ± 29.3%, respectively, with both values exceeding the discriminate cutoff values in the aforementioned studies. Although these studies all demonstrated differences in the SUV_max_ of concomitant tumors, further investigation on the efficacy, accuracy, and the usefulness of the SUV_max_ ratio and DI_SUVmax_ is needed. Some patients show false-positive results for the presence of SMPMNS. Metastasis, premalignant lesion, or benign lesion may be misinterpreted as another primary lesion on ^18^F-FDG PET/CT (25).

**Figure 8:**
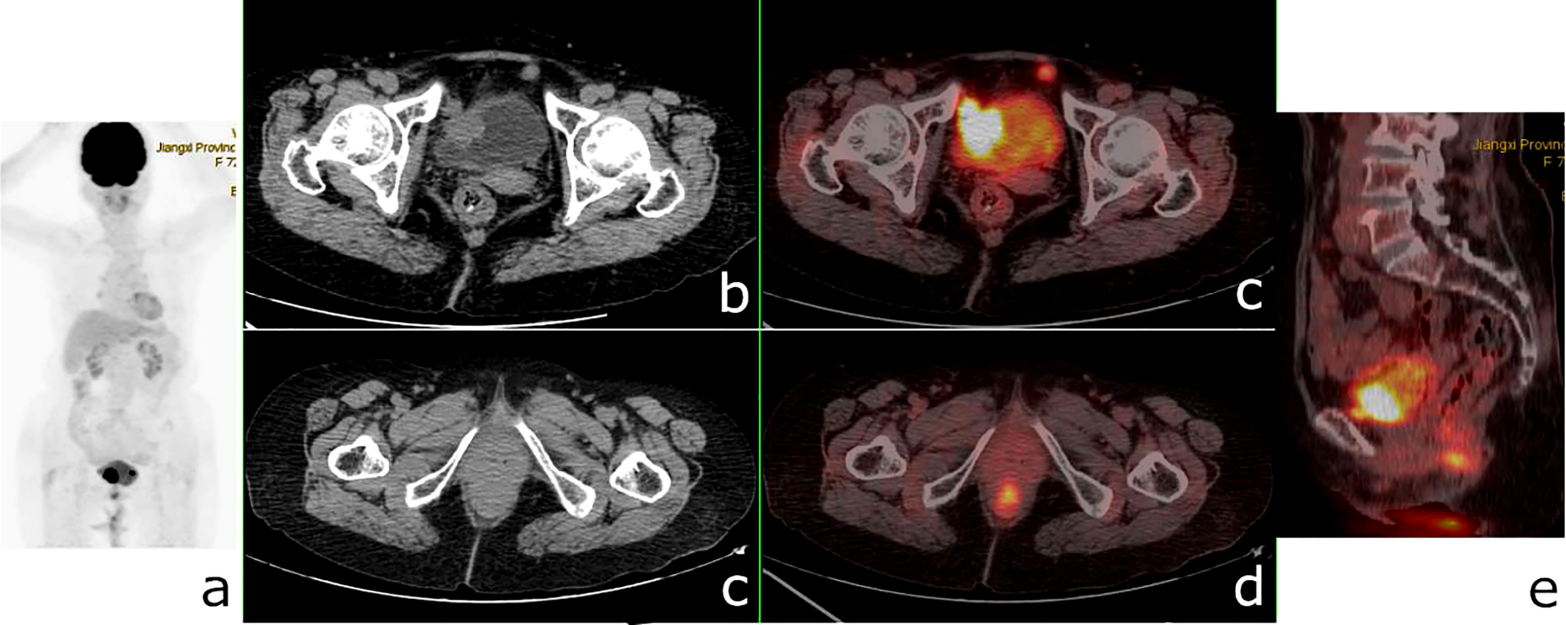
^18^F-fluorodeoxyglucose positron emission tomography/computed tomography (^18^F-FDG PET/CT) of a 72-year-old woman with bladder cancer and anal canal adenocarcinoma. PET/CT revealed a 26 × 24-mm nodule in the bladder wall with a maximum standardized uptake value (SUV_max_) of 23.9, a nodular hypermetabolic lesion (SUV_max_ = 11.3) misinterpreted as physiological FDG uptake in the anal canal, and a small nodule with a diameter of 1 cm in front of the bladder (SUV_max_ = 8.9). **(A)** PET/MIP. **(B, C)** Axial CT. **(C, D)** Fusion images. **(E)** Sagittal fusion image.

**Figure 9:**
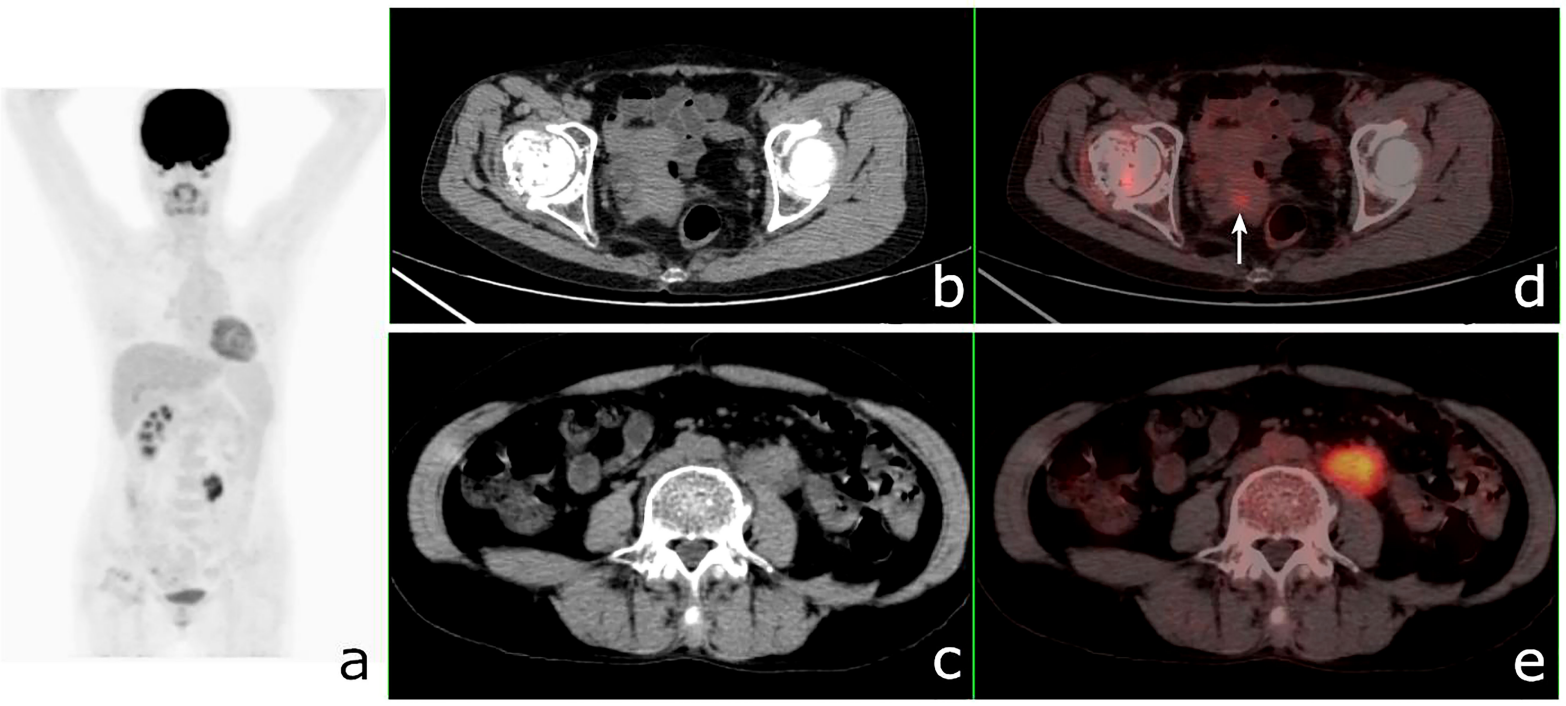
^18^F-fluorodeoxyglucose positron emission tomography/computed tomography (^18^F-FDG PET/CT) of a 49-year-old woman with ureteral squamous cell carcinoma and endometrial carcinoma. PET/CT revealed a 41 × 20-mm mass in the left ureter with a maximum standardized uptake value (SUV_max_) of 7.7 and a nodular hypermetabolic lesion (SUV_max_ = 2.7) (*arrow*) misinterpreted as physiological FDG uptake in the uterine. **(A)** PET/MIP. **(B, C)** Axial CT. **(C, D)** Fusion images.

To our knowledge, there are only a few studies evaluating the diagnostic performance of ^18^F-FDG PET/CT for SMPMNS. In this series, SMPMNS were considered in 28 cases, and most of the primary lesions (including diagnosed and misdiagnosed) have been revealed on ^18^F-FDG PET/CT imaging. We noted one primary tumor of SMPMNS that was more frequently missed on CI primarily due to the regional imaging modalities and the general lack of awareness regarding SMPMNS on the part of clinicians, with their attention tending to be focused on the identified primary lesion. ^18^F-FDG PET/CT, which can more comprehensively reveal lesions due to its whole body surveillance, will help in increasing clinicians’ awareness regarding SMPMNS. Additionally, one primary tumor of SMPMNS was easily mistaken for metastasis, especially in patients with metastases, which will more likely lead to confusion in the diagnosis. ^18^F-FDG PET/CT, which allows combining metabolic information with anatomic details, reduces the incidences of misdiagnosis, i.e., tumors with different clonal origins that were generally believed to have a different biological behavior, leading to different uptakes of FDG. Misinterpretation of a high FDG uptake in lesions as physiological uptake on PET/CT is also an important reason for the missed diagnosis (**Figures 8**, **9**).

The therapeutic regimens and the prognosis of SMPMNS patients with or without metastasis were different. In this series, preoperative PET/CT examination improved the diagnostic accuracy and changed the treatments for some patients. Comparison of the survival times of SMPMNS patients with and without documented metastases showed a significant difference in the overall survival rates between these two categories. In this series, more than one-third of the cases showed suspected metastases on ^18^F-FDG PET/CT, and the patient-based diagnostic accuracy of ^18^F-FDG PET/CT and CI for metastases was not significantly different; nevertheless, ^18^F-FDG PET/CT had higher sensitivity, as it detected more unexpected metastatic lesions that were missed or not imaged on CI. However, ^18^F-FDG PET/CT had lower specificity, as it overestimated the number of metastatic lesions in some cases of SMPMNS, particularly the number of metastatic lymph nodes. Similar findings have been observed in the ^18^F-FDG PET/CT assessments of patients with other malignancies (26). Increasing the SUV_max_ threshold for the diagnosis of lymph node metastasis may improve the diagnostic accuracy (27).

In this series, combination with the serum tumor marker levels may improve the diagnostic accuracy of PET/CT of multiple primary cancers, particularly for a number of suspected tumor patients with other highly specific biomarkers, such as prostate-specific antigen (PSA) and alpha-fetoprotein (AFP). However, most biomarkers are nonspecific; therefore, even if the serum levels of two or more biomarkers are elevated, there is still a limitation in the diagnosis of synchronous multiple primary cancers based on these markers (**Supplementary Table S1**).

Indeed, the diagnosis of SMPMNs is quite difficult due to the uniqueness of each tumor, and ^18^F-FDG PET/CT can sometimes also fail to reach a definite diagnosis. Different radiotracers can reveal varied biological characteristics of different tumors, and they have currently been applied in the diagnosis of SMPMNS (28, 29). The stepwise application of different radiotracers for the diagnosis of SMPMNS may have a broad prospect; however, it will also impose an additional economic burden on patients.

The main limitation of this study is its small sample size due to rarity of SMPMNS. Additionally, selective bias and data bias were inevitable because all of patients in this study were from a single hospital.

In conclusion, SMPMNS are mostly double primary tumors that generally occur in the elderly, and the lung is the most common primary tumor site. Different primary tumors usually show differences in the uptake of FDG. When combined with clinical features, ^18^F-FDG PET/CT can improve the diagnostic performance of SMPMNS and can reveal more primary tumors and metastatic lesions. It is helpful to increasing the awareness of clinicians regarding SMPMNS and reduces the number of missed diagnosis and misdiagnosis. For patients with SMPMNS, ^18^F-FDG PET/CT is a good imaging modality.

## Data availability statement

The original contributions presented in the study are included in the article/**Supplementary Material**. Further inquiries can be directed to the corresponding author.

## Ethics statement

The study was conducted in accordance with the Declaration of Helsinki (as revised in 2013). The study was approved by the Institutional Ethics Committee of Jiangxi Provincial People’s Hospital, and individual consent for this retrospective analysis was waived.

## Author contributions

Luo ZH: Conception and design. Luo ZH and Jin AF: Administrative support. Luo ZH, Qi WL, Liao FX and Liu Q: Provision of study materials or patients and data analysis and interpretation. Luo ZH, Qi WL, Jin AF, and Zeng QY: Collection and assembly of data. All authors wrote the manuscript. All authors contributed to the article and approved the submitted version. 

## Funding

This work was funded by the Science-Technology Supporting Projects of Jiangxi Sci-Tech Department (20142BBG70095) and the Science and Technology Plan of Jiangxi Provincial Health Commission (202130087).

## Conflict of interest

The authors declare that the research was conducted in the absence of any commercial or financial relationships that could be construed as a potential conflict of interest.

## Publisher’s note

All claims expressed in this article are solely those of the authors and do not necessarily represent those of their affiliated organizations, or those of the publisher, the editors and the reviewers. Any product that may be evaluated in this article, or claim that may be made by its manufacturer, is not guaranteed or endorsed by the publisher.
