# Supplementary material for: The role of 18F-FDG PET/CT in patients with synchronous multiple primary malignant neoplasms occurring at the same time
Source: Front Oncol. 2022 Dec 2;12:1068055. doi: 10.3389/fonc.2022.1068055 (PMC9757168; doi:10.3389/fonc.2022.1068055)
Supplement: Supplementary file 1 [file Table_1.docx]

**Supplementary table 1:** Serum level of biomarker before PET/CT imaging in 37 patients with SMPMNS

| **case** | **biomarker** | | | | | | | |
| --- | --- | --- | --- | --- | --- | --- | --- | --- |
|  | **CEA** | **PSA** | **CA19-9** | **CA125** | **Ca153** | **SCC** | **CYFRA 21-1** | **AFP** |
| **1** | **n** | **-** | **n** | **n** | **n** | **-** | **-** | **-** |
| **2** | **↑** | **-** | **n** | **-** | **-** | **-** | **-** | **-** |
| **3** | **n** | **↑** | **n** | **-** | **-** | **-** | **n** | **-** |
| **4** | **↑** | **-** | **n** | **-** | **-** | **-** | **n** | **-** |
| **5** | **↑** | **-** | **n** | **-** | **-** | **-** | **n** | **↑** |
| **6** | **↑** | **-** | **n** | **-** | **-** | **-** | **n** | **-** |
| **7** | **n** | **↑** | **↑** | **-** | **-** | **n** | **n** | **-** |
| **8** | **↑** | **-** | **n** | **-** | **-** | **-** | **n** | **-** |
| **9** | **n** | **-** | **n** | **↑** | **↑** | **-** | **↑** | **-** |
| **10** | **n** | **-** | **n** | **n** | **-** | **-** | **-** | **-** |
| **11** | **↑** | **-** | **n** | **-** | **-** | **n** | **-** | **-** |
| **12** | **n** | **-** | **n** | **-** | **-** | **↑** | **-** | **-** |
| **13** | **n** | **-** | **n** | **-** | **-** | **-** | **n** | **-** |
| **14** | **↑** | **-** | **n** | **n** | **n** | **-** | **↑** | **-** |
| **15** | **n** | **-** | **n** | **-** | **-** | **-** | **-** | **-** |
| **16** | **n** | **-** | **n** | **-** | **-** | **n** | **↑** | **-** |
| **17** | **↑** | **-** | **n** | **-** | **-** | **n** | **n** | **-** |
| **18** | **↑** | **-** | **n** | **-** | **-** | **-** | **n** | **-** |
| **19** | **↑** | **-** | **n** | **-** | **-** | **-** | **-** | **-** |
| **20** | **n** | **-** | **n** | **-** | **-** | **-** | **-** | **-** |
| **21** | **n** | **-** | **n** | **-** | **-** | **-** | **n** | **-** |
| **22** | **n** | **-** | **n** | **-** | **-** | **n** | **-** | **-** |
| **23** | **↑** | **-** | **↑** | **n** | **-** | **-** | **-** | **-** |
| **24** | **n** | **↑** | **n** | **-** | **-** | **-** | **-** | **-** |
| **25** | **n** | **-** | **n** | **-** | **-** | **-** | **n** | **-** |
| **26** | **n** | **-** | **n** | **-** | **-** | **-** | **n** | **-** |
| **27** | **↑** | **-** | **n** | **-** | **-** | **-** | **n** | **-** |
| **28** | **↑** | **-** | **↑** | **-** | **-** | **-** | **n** | **-** |
| **29** | **n** | **↑** | **n** | **-** | **-** | **-** | **-** | **-** |
| **30** | **n** | **-** | **n** | **-** | **-** | **-** | **n** | **-** |
| **31** | **↑** | **-** | **↑** | **-** | **-** | **-** | **n** | **-** |
| **32** | **↑** | **-** | **n** | **-** | **-** | **-** | **n** | **-** |
| **33** | **↑** | **-** | **↑** | **-** | **-** | **-** | **n** | **-** |
| **34** | **n** | **-** | **n** | **-** | **-** | **-** | **n** | **-** |
| **35** | **n** | **-** | **n** | **-** | **-** | **-** | **n** | **-** |
| **36** | **n** | **-** | **-** | **-** | **-** | **-** | **n** | **-** |
| **37** | **↑** | **-** | **n** | **-** | **-** | **-** | **-** |  |

Note: SMPMNS, synchronous multiple primary malignant neoplasm at the same time. n, within the normal range;

-, not detected. The case numbers are the same as table 1.
